# Exploratory Analysis of Candidate Gene Variants in Developmental Dysplasia of the Hip: Evidence for the Role of GDF5 rs143384

**DOI:** 10.3390/genes17020129

**Published:** 2026-01-25

**Authors:** Stefan Harsanyi, Lucia Neuschlova, Lubica Milosovicova, Radoslav Zamborsky, Andrea Pastorakova, Lubos Danisovic

**Affiliations:** 1 Institute of Medical Biology, Genetics and Clinical Genetics, Faculty of Medicine, Comenius University in Bratislava, Sasinkova 4, 811 08 Bratislava, Slovakia; schwarzova27@uniba.sk (L.N.); lubica.milosovicova@fmed.uniba.sk (L.M.); andrea.pastorakova@fmed.uniba.sk (A.P.); lubos.danisovic@fmed.uniba.sk (L.D.); 2 Orthopaedic Clinic, Faculty of Medicine, Comenius University in Bratislava, Limbova 1, 833 40 Bratislava, Slovakia; radozamborsky@gmail.com

**Keywords:** developmental dysplasia of the hip, DDH, gene polymorphism, GDF5, CX3CR1, MLPA, CNV

## Abstract

Background: Developmental dysplasia of the hip (DDH) is a common orthopedic disorder characterized by abnormal development of the hip joint, which can lead to pain, instability, and early-onset osteoarthritis if left untreated. Its etiology is multifactorial, involving both genetic and environmental factors. Methods: This study investigated the association between selected single-nucleotide polymorphisms (SNPs) related to joint and bone development and the occurrence of DDH. It assessed potential copy number variations (CNVs) in key skeletal genes using MLPA. A total of 125 individuals were examined, including 43 patients with DDH and 82 healthy controls. Six SNPs were genotyped using real-time PCR with TaqMan assays: *TGFB1* (rs1800470), *CX3CR1* (rs3732378, rs3732379), *GDF5* (rs143384), *COL1A1* (rs113647555), and *MMP24* (rs12479765). Allele and genotype distributions were compared between cases and controls, and CNVs in *COL1A1*, *COL2A1*, *LRP5*, *DKK1*, *FZD4*, and *NDP* genes were analyzed using Multiplex Ligation-Dependent Probe Amplification. Results: Among the examined variants, only GDF5 rs143384 showed a nominally significant association with DDH (*p* = 0.040), with the A allele more common in affected individuals. However, after correcting for multiple testing, this result no longer remained significant. No significant associations were detected for *TGFB1*, *CX3CR1*, *COL1A1*, or *MMP24*. Although *CX3CR1* rs3732378 allele frequencies differed slightly from international reference data, no link to DDH was confirmed. Conclusions: MLPA analysis did not identify pathogenic CNVs in the analyzed loci, which indicates that the studied genes have no association with DDH in the Slovak population. Similarly, SNPs in the studied genes yielded no significant results, apart from rs143384 in *GDF5*, which requires further investigation to confirm our findings.

## 1. Introduction

Developmental dysplasia of the hip (DDH) is a common congenital musculoskeletal disorder that affects the shape and stability of the hip joint, and, if untreated, can result in gait abnormalities, chronic pain, and early-onset osteoarthritis [1]. The global incidence of DDH shows considerable variation by ethnicity and geography, ranging from 0.06 per 1000 live births in African populations to 76.1 per 1000 in Native Americans [2]. In Japan, the national incidence of DDH-related dislocation is 0.076%, whereas in the United Kingdom, it is approximately 4.9 per 1000 live births [3]. A large cohort study in China identified an incidence of 1.7% among infants [4]. Across Europe, DDH incidence in neonates ranges from 0.8 to 27.5 per 1000 live births, with substantial intra-country variation [5,6,7].

DDH is much more common in females, with female-to-male ratios as high as 7:1. The left and unilateral forms are the most common, but bilateral involvement also occurs [8,9]. DDH is a multifactorial disease with both genetic predisposition and environmental influence. Significant risk factors include a family history, breech presentation, primiparity, high birth weight, and post-maturity, whereas low birth weight and prematurity are protective factors [10]. Environmental influences, such as tight swaddling and seasonal variation at birth, also increase risk, as do associated conditions, such as oligohydramnios or congenital foot deformities [11,12]. A genetic origin is increasingly recognized, with several candidate genes implicated in the development of the joints, the architecture of connective tissues, and in extracellular matrix remodeling [13,14]. Despite these observations, the functional mechanisms by which specific polymorphisms act to influence hip joint development remain poorly defined and thus warrant focused genetic studies.

In this study, we investigated six single-nucleotide polymorphisms (SNPs) previously studied across multiple countries in relation to DDH. *TGFB1* (rs1800470) encodes transforming growth factor-β1, a key regulator of chondrogenesis, osteoblast differentiation, and extracellular matrix production [15]. The rs1800470 was associated with DDH in a large Han-Chinese cohort, whereas a Turkish study did not replicate this finding [16,17]. Functionally, altered TGF-β1 signaling may influence acetabular cartilage development and joint stability. *CX3CR1* (rs3732378, rs3732379) encodes a chemokine receptor involved in inflammatory signaling and bone remodeling [18]. One case-control study reported an increased risk of DDH for both SNPs, but another study found no association, indicating conflicting evidence [19,20,21]. Variants in *CX3CR1* may modulate chondrocyte response to mechanical stress by altering chemokine-mediated pathways. GDF5 (rs143384) is a key morphogen for limb and joint development [22]. GWAS has implicated this variant/locus in DDH. Functional work demonstrates reduced *GDF5* expression for the linked SNP rs143383, with rs143384 in cis modulating this effect [22,23]. Lower GDF5 expression may impair normal hip joint morphogenesis. *COL1A1* (rs113647555) encodes the α1 chain of type I collagen. Its variants may affect ligamentous laxity. A small study reported promoter variants (including rs113647555) in DDH cases versus controls, but larger confirmatory studies are lacking [24]. *MMP24* (rs12479765) encodes a matrix metalloproteinase implicated by GWAS in DDH [22]. The causal mechanism in DDH remains unclear, but dysregulation may influence acetabular shaping and soft-tissue stability.

In addition to SNP genotyping, we performed Multiplex Ligation-Dependent Probe Amplification (MLPA) to detect possible copy number variations (CNVs) in the *COL1A1*, *COL2A1*, *LRP5*, *DKK1*, *FZD4*, and NDP genes. *COL1A1* and *COL1A2* exon deletions have been reported in arthrochalasia type Ehlers–Danlos syndrome, a rare connective tissue disorder characterized by congenital bilateral hip dislocation, representing a syndromic rather than idiopathic form of DDH [25]. No CNVs in *COL2A1*, *LRP5*, *DKK1*, *FZD4*, or *NDP* have been associated with DDH to date. Therefore, we also employ this method to verify CNV status in non-syndromic DDH individuals.

The aim of this study was to examine the association between DDH and six selected SNPs in a Slovak Caucasian population and to screen for CNVs in key genes involved in bone development using MLPA. By combining SNP and MLPA approaches, we aimed to expand the genetic understanding of DDH, identify potential molecular markers for diagnosis or risk prediction, and assess their contribution in a Central European cohort.

## 2. Materials and Methods

### 2.1. Study Population

The study included 125 participants, consisting of 43 patients diagnosed with developmental dysplasia of the hip (DDH) and 82 healthy controls. The DDH group comprised 37 females (86%) and six males (14%), while the control group included 50 females (61%) and 32 males (39%). Participants were from various regions of Slovakia. All participants were examined at the Orthopedic Clinic of the Medical Faculty of Comenius University and the National Institute of Children’s Diseases in Bratislava between 2018 and 2022. To ensure the predominance of osteogenic etiology, only individuals with isolated DDH were enrolled. Patients with systemic diseases, syndromic diagnoses, generalized skeletal dysplasias, or connective tissue disorders were excluded from the study. Patient histories did not indicate any environmental contribution in the study group. Family members of patients with a family history of DDH were not included in the study. Detailed personal and family medical histories were collected for all participants. Control individuals were selected based on the absence of DDH or other musculoskeletal disorders in both personal and family history. Additionally, hip ultrasound (USG) examinations conducted during infancy confirmed no signs of DDH in the control group. Participants were recruited from various regions of Slovakia. The study was conducted in accordance with the 2013 Declaration of Helsinki. It was approved by the Ethics Committee of the National Institute of Children’s Diseases in Bratislava (VEGA 1/0255/17). Written informed consent was obtained from all participants or their legal guardians.

### 2.2. Genotyping

Peripheral blood samples were collected from all participants. Genomic DNA was extracted using the GeneJET Genomic DNA Purification Kit (Thermo Fisher Scientific, Waltham, MA, USA) according to the manufacturer’s instructions. Genotyping was performed using assays presented in Table 1.

Genotyping of selected SNPs was performed using pre-designed TaqMan SNP Genotyping Assays (Thermo Fisher Scientific, Waltham, MA, USA) on a QuantStudio 3 Real-Time PCR System (Applied Biosystems, Thermo Fisher Scientific, Waltham, MA, USA). Each reaction was prepared in a total volume of 15 µL, consisting of 7.5 µL of 2× Maxima Probe/ROX qPCR Master Mix (Thermo Fisher Scientific, Waltham, MA, USA), 0.75 µL of 20× TaqMan SNP Genotyping Assay (containing allele-specific primers and fluorescently labeled probes), 5.25 µL of nuclease-free water, and 1.5 µL of genomic DNA (~20 ng).

The 20× working solution of the TaqMan assay was prepared by diluting the original 40× Probe + Primer Mix 1:1 with nuclease-free water. For each reaction, 13.5 µL of premixed master mix (excluding DNA) was aliquoted into wells, followed by the addition of 1.5 µL of sample DNA. No-template controls (NTCs) were included in each run by substituting DNA with nuclease-free water to monitor contamination and assay specificity. Thermal cycling was performed under the following conditions: Initial denaturation at 95 °C for 10 min, followed by 40 cycles of denaturation at 95 °C for 15 s and annealing and extension at 60 °C for 1 min. All genotyping reactions and post-PCR analyses were carried out at the Institute of Medical Biology, Genetics, and Clinical Genetics, Faculty of Medicine, Comenius University in Bratislava.

Each TaqMan genotyping run included positive controls consisting of samples with genotypes previously confirmed by next-generation sequencing, as well as a no-template negative control to monitor potential contamination. No repeated samples were required because the assays showed consistent amplification curves and allele clustering across plates. Genotyping was successful in 125 of 126 samples, yielding a success rate of 99.2%. The single failed reaction was excluded.

### 2.3. MLPA Analysis

Multiplex Ligation-Dependent Probe Amplification was performed using three SALSA^®^ MLPA^®^ probemixes: P271-B5 COL1A1, P214-C2 COL2A1, and P285-C3 LRP5, following the manufacturer’s protocol (MRC-Holland, Amsterdam, The Netherlands). The MLPA reactions were carried out to assess copy number variations in selected exons of the COL1A1, COL2A1, LRP5, DKK1, FZD4, and NDP genes. PCR amplification products were separated and detected using the SeqStudio™ 8 Flex Genetic Analyzer (Applied Biosystems, Life Technologies, Waltham, MA, USA). Data analysis and probe signal normalization were performed using Coffalyser.Net software (version 220513.1739; MRC-Holland, Amsterdam, The Netherlands).

The P271-B5 probemix for COL1A1 includes 33 probes targeting 33 of the gene’s 51 exons. The P214-C2 probemix for COL2A1 contains 46 probes covering 43 different exons out of the total 54. The P285-C3 probemix includes probes for multiple genes: 24 probes covering all 23 exons of LRP5, four probes for each of the four exons of DKK1, three probes targeting the two exons of FZD4, and three probes covering all three exons of NDP. These combined assays allowed for comprehensive evaluation of potential exon-level deletions or duplications in key genes associated with bone and connective tissue disorders. This was the only available kit to detect CNVs in connective tissue-related disorders.

### 2.4. Statistical Analysis

Allele and genotype frequencies were calculated for each SNP. Comparisons between the developmental DDH group and controls were performed using Pearson’s chi-square test. Genotype distributions were tested for compliance with the Hardy-Weinberg equilibrium (HWE) in the control group and compared with expected frequencies reported for the Caucasian population. To account for multiple comparisons, *p*-values were adjusted using the Bonferroni correction (α = 0.05/6 = 0.0083). Both raw and adjusted *p*-values are reported. Statistical significance was defined as *p* < 0.05 before correction, and as *p* < 0.0083 after Bonferroni adjustment. A post hoc power analysis was performed using the observed allele frequencies and sample sizes to estimate the study’s ability to detect moderate genetic effects. All statistical analyses were conducted using IBM SPSS Statistics version 29.0 (IBM Corp., Armonk, NY, USA).

## 3. Results

In the study, 125 samples were tested for multiple SPNs (43 subjects and 82 controls). Within the DDH group, affection of the left hip was observed in 33 cases (76.7%), right hip in 7 cases (16.3%), and bilateral in 3 cases (7.0%). A positive family history of DDH was reported in 14 patients (32.6%). Only individuals with isolated DDH were included in the study; patients with systemic, syndromic, or generalized skeletal disorders were excluded. All controls had no clinical, imaging, or family history suggestive of DDH.

A comparison of observed allelic frequencies for the six investigated SNPs with international population databases is provided in Table 2. For all SNPs, the pooled allele frequencies observed in our Slovak cohort were generally consistent with those reported for European populations (HapMap, 1000 Genomes, and Applied Biosystem databases). The genotype distributions were in Hardy-Weinberg equilibrium in both the case and control groups for all loci.

We observed no statistically significant differences in allele distribution between subjects with DDH and reference Caucasian/European populations. The frequencies of all studied genes except for GDF5 were comparable to those reported in the 1000 Genomes European dataset, with all *p*-values > 0.05. GDF5 rs143384 showed a modest deviation from HapMap and 1000 Genomes. These findings suggest that the allele distributions in our study population are consistent with broader European genetic backgrounds.

In Table 3, we present the distribution of genotypes and alleles regarding the studied SNPs. Our analysis revealed statistically significant changes in the distribution for rs143384 in GDF5 (*p* = 0.040). Individuals with DDH showed a higher frequency of the A allele and AA genotype compared with controls. In the allelic model (A vs. G), the odds ratio was 1.93 (95% CI 1.12–3.32), and in the recessive model (AA vs. GA+GG), the odds ratio was 2.39 (95% CI 1.08–5.28). No other associations between genotype distributions and DDH status were found for the remaining studied SNPs. The remaining polymorphisms were in HWE in both the subject and control groups.

Using the MLPA method, we analyzed copy-number variation (CNV) in individual selected genes and their exons, which we assumed could play a role in DDH. In only one case out of 43 tested patients with DDH, we did find a CNV of *COL1A1* exon 38 duplication.

None of the identified associations remained statistically significant after correction for multiple testing (Bonferroni α = 0.0083). There is therefore no consistent evidence at the current sample size, supporting a significant relationship between the examined SNPs and the risk of DDH. However, the *GDF5* rs143384 variant may be a potentially suggestive locus that deserves further investigation in larger cohorts.

A post hoc power analysis based on observed allele frequencies and sample sizes (43 cases; 82 controls) indicates that this study had only moderate power to detect a moderate genetic effect (OR ≈ 2.0) in the common variants, with power ranging from ~60–75% at α = 0.05 and ~35–50% after Bonferroni correction. For the rare *COL1A1* variant, power was <10%. Based on these findings, this study is underpowered to detect small effects or rare-variant associations.

## 4. Discussion

In this study, we examined six genetic variants—TGFB1 rs1800470, CX3CR1 rs3732378 and rs3732379, GDF5 rs143384, COL1A1 rs113647555, and MMP24 rs12479765—in a Slovak Caucasian cohort comprising 43 DDH patients and 82 healthy controls. Overall, our findings revealed no significant association between DDH and CX3CR1, TGFB1, COL1A1, or MMP24 polymorphisms at the allele or genotype level. Only GDF5 rs143384 demonstrated a nominally significant difference (*p* = 0.040), suggesting a possible contribution to DDH predisposition, although the value did not remain statistically significant after correction for multiple testing.

Our cohort’s allele frequencies for all six loci aligned well with European reference data from HapMap, 1000 Genomes, and Applied Biosystems, confirming the representativeness of our sample. The exception was CX3CR1 rs3732378, which showed a significant difference in allele distribution compared to reference datasets (*p* < 0.001), though it was not associated with DDH. This may reflect population-specific variation or sampling heterogeneity rather than a causal relationship.

Our results on CX3CR1 align with a study by Qiao et al. of Han Chinese patients, who reported no association between rs3732378 or rs3732379 and DDH in a Han Chinese cohort [21]. In contrast, Li et al. (2017) observed significant associations in a larger Chinese sample, where the rs3732378 minor allele increased DDH risk (OR = 2.25, *p* = 0.003) and rs3732379 showed a similar effect (OR = 1.84, *p* = 0.017) [20]. Gumus et al. similarly reported a significant association of rs3732378 in the Turkish population [26]. These discrepancies likely reflect differences in ethnic background, allele frequency, and sample size across studies. At the experimental level, Feldman et al. demonstrated that Cx3cr1-knockout mice develop acetabular enlargement and gait abnormalities reminiscent of hip instability, supporting a developmental role for CX3CR1 signaling [18]. Nevertheless, our human data do not indicate a significant contribution of CX3CR1 polymorphisms to DDH in the Slovak population.

The most consistent evidence of genetic association was observed for GDF5 rs143384, a variant repeatedly linked to DDH across populations. Genome-wide association studies from the UK Biobank (OR = 1.44, *p* = 3.55 × 10^−22^) and independent cohorts from France (OR = 1.53, *p* = 0.002) and Brazil (AA genotype 32% vs. 14%, *p* = 0.01) have confirmed its involvement [27,28]. GDF5 encodes a member of the bone morphogenetic protein family that regulates chondrogenesis and joint morphogenesis. Therefore, its biological role provides a mechanistic basis for its influence on hip development. Several studies also implicate rs143383, another GDF5 promoter variant, in DDH susceptibility [29,30,31].

Our negative results for COL1A1 are consistent with other European investigations reporting no association with DDH [32]. Similarly, no significant effects were found for TGFB1 or MMP24, in contrast to reports from Asian cohorts suggesting possible involvement [15,22]. Ethnic differences, gene–environment interactions, and limited sample sizes may explain these discrepancies.

CNV analysis using MLPA detected a single duplication of exon 38 in the COL1A1 gene among 43 DDH patients. No CNVs were found in COL2A1 or LRP5, indicating that large-scale genomic rearrangements in these loci are rare and unlikely to represent a major pathogenic mechanism in DDH.

Taking these data together, our study confirms previous evidence implicating GDF5 rs143384 as a candidate risk variant for DDH and suggests that CX3CR1, TGFB1, COL1A1, and MMP24 are unlikely to play a significant role in disease susceptibility in the Slovak population. The findings emphasize the polygenic nature of DDH and the requirement for larger, multi-ethnic studies to clarify both the contribution of individual loci and their interaction with developmental and biomechanical factors. Clinically, these results imply that common variants of the type investigated here have limited predictive value for DDH risk assessment in this population, and only the modest, uncorrected association at GDF5 rs143384 would warrant further evaluation.

This study has several limitations. The most important is the relatively small sample size, particularly within the DDH group, which reduces statistical power to detect modest genetic effects. The underrepresentation of male subjects further limits the robustness of sex-stratified analyses and may affect the generalizability of the findings. Additionally, although the homogeneous Slovak-Caucasian background minimizes confounding due to population stratification, it limits the applicability of these results to other ethnic groups. Finally, the cross-sectional design precludes functional or mechanistic inference regarding how the identified variants contribute to DDH pathogenesis.

## 5. Conclusions

In conclusion, this study found no strong evidence of association between DDH and polymorphisms in CX3CR1, TGFB1, COL1A1, or MMP24. The GDF5 rs143384 emerged as a nominally significant and biologically plausible candidate variant but still needs verification in larger studies. These findings are consistent with prior evidence suggesting that GDF5 plays a role in hip joint morphogenesis and DDH susceptibility. MLPA analysis did not yield any significant results.

Future research should focus on larger, multi-ethnic cohorts and integrate multiomic and functional genomic approaches to elucidate the regulatory and epigenetic mechanisms underlying GDF5-related developmental pathways. Longitudinal and developmental-stage–specific analyses may further clarify how genetic variants interact with mechanical, hormonal, and environmental factors during acetabular and femoral head formation.

## Figures and Tables

**Table 1:** TaqMan assays used.

SNP (Gene)	Assay ID	Alleles (VIC/FAM)	Substitution
*rs1800470* (*TG* *FB1*)	C_22272997_10	A (VIC, 50%)/G (FAM, 49%)	G > A
*rs3732378* (*CX3CR1*)	C_5687_1_	A (VIC, 16%)/G (FAM, 84%)	G > A
*rs3732379* (*CX3CR1*)	C_7900503_1_	C (VIC, 73%)/T (FAM, 26%)	C > T
*rs143384* (*GDF5*)	C_599144_1_	A (VIC, 55%)/G (FAM, 44%)	G > A
*rs113647555* (*COL1A1*)	C_25474005_10	A (VIC, 1%)/G (FAM, 98%)	G > A
*rs12479765* (*MMP24*)	C_1271650_20	A (VIC, 19%)/G (FAM, 80%)	G > A

**Table 2:** Comparison of allelic frequencies between the control group in our study and international databases.

Gene	RefSNP	Alleles	Allele Frequencies			
			Our Study (Caucasian)	HapMap (CEU)	1000 Genome (EUR)	Applied Biosystems (Caucasian)
*TGFB1*	rs1800470	G/A	0.41/0.59	N/A	0.38/0.62	N/A
*CX3CR1*	rs3732378	A/G	0.20/0.80	0.17/0.83	0.17/0.83	0.17/0.83
*CX3CR1*	rs3732379	T/C	0.29/0.71	0.26/0.74	0.29/0.71	0.28/0.72
*GDF5*	rs143384	G/A	0.48/0.52	0.36/0.64	0.41/0.59	0.46/0.54
*COL1A1*	rs113647555	G/A	0.99/0.01	N/A	0.99/0.01	N/A
*MMP24*	rs12479765	G/A	0.80/0.20	0.81/0.19	0.84/0.16	N/A

**Table 3:** The distribution of genotypes in the study group and controls.

		Subjects			Controls			χ2 *p*-Value
Gene		Male	Female	All	Male	Female	All	
*TGFB1* rs1800470 G→A	GG	0	5	5	2	8	10	0.498 (OR = 1.21; 95% CI 0.73–2.00)
	GA	3	18	21	22	26	48	
	AA	3	14	17	8	16	24	
*CX3CR1* rs3732378 G→A	GG	2	24	26	24	26	50	0.896 (OR = 1.07; 95% CI 0.63–1.82)
	GA	4	12	14	8	23	31	
	AA	0	1	1	0	1	1	
*CX3CR1* rs3732379 C→T	CC	2	19	21	19	21	40	0.527 (OR = 0.96; 95% CI 0.59–1.59)
	CT	2	15	17	11	26	37	
	TT	2	3	5	2	3	5	
*GDF5* rs143384 G→A	GG	1	2	3	10	6	16	0.040 (OR = 1.93; 95% CI 1.12–3.32)
	GA	4	18	22	15	32	47	
	AA	1	17	18	7	12	19	
*COL1A1* rs113647555 G→A	GG	6	37	43	32	49	81	0.467 (OR not meaningful)
	GA	0	0	0	0	1	1	
	AA	0	0	0	0	0	0	
*MMP24* rs12479765 G→A	GG	4	27	31	18	34	52	0.527 (OR = 0.77; 95% CI 0.43–1.39)
	GA	2	8	10	13	14	27	
	AA	0	2	2	1	2	3	

## Data Availability

The raw data supporting the conclusions of this article will be made available by the authors on request.
